# The Role of Musical Aesthetic Emotions in Social Adaptation to the Covid-19 Pandemic

**DOI:** 10.3389/fpsyg.2021.611639

**Published:** 2021-03-12

**Authors:** Pietro Sarasso, Irene Ronga, Marco Neppi-Modona, Katiuscia Sacco

**Affiliations:** BIP (BraIn Plasticity and Behaviour Changes) Research Group, Department of Psychology, University of Turin, Turin, Italy

**Keywords:** Covid-19, cognitive dissonance, uncertainty, music, neuroaesthetics, emotions, social change, aesthetic appreciation

## Introduction

During the coronavirus disease 2019 (Covid-19) pandemic lockdown, people from all over the world shared all sorts of artistic experiences. Singing or playing music at the window is just a few examples of artistic activities that kept us feeling “together” while being apart. Why did we choose artistic expression, and especially music, to communicate with others during the quarantine? Should we regard this behavior only as a distraction, or is it possible that music evolved as a social tool to help us, perhaps unconsciously, to adapt in times of social distress? In other words, can music be considered as an adaptive form of artistic expression? Here, we will suggest that this may indeed be the case.

In times of greater uncertainty brought by unpredicted situations, such as those we lived during the Covid-19 outbreak (Baker et al., 2020), we are sometimes pushed to reduce our discomfort by conservatively dismissing alternative behaviors and avoiding information that disconfirms our previously acquired beliefs, behaviors, or cognitions. The tension produced by inter- or intrapersonally inconsistent (i.e., ambiguous) thoughts, attitudes, perceptions, or behaviors is what Festinger (1957) referred to as “cognitive dissonance.” Interestingly, the concept of cognitive dissonance has informed the public debate around lockdown measures and health policies in the USA during the Covid-19 pandemic. One of the founders of the cognitive dissonance theory, the social psychologist Elliot Aronson, commented that interpersonal cognitive dissonance might be “the motivational mechanism that underlies the reluctance to admit mistakes or accept scientific findings” (Aronson and Tavris, 2020). This aversive psychological drive triggers a series of dissonance reduction strategies (e.g., act rationalization, behavioral change, and denial of responsibility, trivialization) that can lead to either attitudinal change or attitudinal bolstering (Cancino-Montecinos et al., 2020). Reducing dissonance by rigidly dismissing alternatives can be potentially detrimental for both individual choices and behaviors as well as for the collective policy (Brady et al., 1995; Margolis et al., 2016). Conversely, the ability to adaptively modify our behavior to unexpected and surprising events is a fundamental human evolutionary conquest. However, during the pandemic, such an adaptive response required us “to live with uncertainty, […] which involves living with the dissonance for a while rather than jumping immediately to a self-justification” (Aronson and Tavris, 2020). Recent accounts of cognitive dissonance (Kaaronen, 2018), framed within the predictive coding theory (Friston, 2010), associate our motivation for “dissonance reduction” (Festinger, 1957) with “prediction error reduction” (Friston, 2010), which, as we will explain thereinafter, also plays an important role in the perceptual, aesthetic, and emotional dimensions of musical experience (Quiroga-Martinez et al., 2019). Prediction errors are transient states of uncertainty induced by mismatches between novel information and preexisting beliefs. The brain actively minimizes prediction errors either by adapting the sensory environment to our representation through (physical or mental) action or through learning by adapting our representations to the sensory environment (Friston, 2010). The balance in the trade-off between these two possibilities determines dissonance reduction strategies (Kaaronen, 2018) and, as we will explain in the following paragraphs, can be influenced by the aesthetic attitude induced by musical experiences (Sarasso et al., 2020a).

As Festinger (1957) himself argued, social communication is a source of cognitive dissonance as well as a vehicle for reducing it (Matz and Wood, 2005). Here, we will review theories suggesting that the appreciation, production, and sharing of music might help individuals and societies to tolerate uncertainty and disturbing emotions, reduce cognitive dissonance in an adaptive way, and learn from the ever-changing environment. In other words, the aesthetic emotions prompted by music might improve and intensify communication, thereby allowing the emergence of collective strategies to reduce dissonance.

## Tolerating Dissonant Uncertainty: Beauty and Knowledge Acquisition

The relation between aesthetic emotions and learning/knowledge acquisition stems from the classical philosophical tradition. In his oeuvre *Poetics*, which is considered as a “learning and inference doctrine” (Tracy, 1946), Aristotle affirms: “The reason in delight in seeing a picture is that one is at the same time learning–gathering the meaning of things” (Tracy, 1946, p. 1). More recently, the aesthetic experience has been described as a cognitive process enhancing the attention toward the beautiful percept (Marković, 2012) and thus supporting the neglect of self-referred concerns (i.e., the Kantian notion of *disinterested interest*). This notion, later reformulated by Schopenhauer as a “will-less” mental state during aesthetic experiences, still influences recent developments in neuroaesthetics (Chatterjee and Vartanian, 2016). Similar interpretations of aesthetic experiences are also found in neuroaesthetic studies of music. Brattico and Pearce (2013) define an aesthetic experience of music “as one in which the individual immerses herself in the music, dedicating her attention to perceptual, cognitive and affective interpretation based on the formal properties of the perceptual experience.”

In our view, the hypothesized ability of aesthetic experience to transitorily free the beholders from “wanting” (Chatterjee and Vartanian, 2014; Kirsch et al., 2016) supports the re-orienting of attention toward knowledge acquisition (Menninghaus et al., 2017; Sarasso et al., 2020a). In our view, such an “aesthetic attitude” (Stolnitz, 1978) is fundamental in order to accept newly acquired knowledge and to update desired states in an ever-changing environment while embracing potentially disturbing or threatening novel sensations and emotions (Sarasso et al., 2020a). Aesthetic emotions might be fundamental to drive our ability to attune with reality and to fully embrace the “here and now” of perception (Menninghaus et al., 2017), an attitude that musicologists define as “openness to experience” (Mencke et al., 2019).

Interestingly, in agreement with the above-mentioned philosophical debate, recent experimental research has suggested that music might serve as a social tool to tolerate cognitive dissonance, thereby helping individuals to adapt (Masataka and Perlovsky, 2012, 2013; Perlovsky, 2015). For example, a study involving 4-year-old children, by Masataka and Perlovsky (2012), showed that participants devalued a toy they were not allowed to play with. Interestingly, music exposure prevented this devaluation. Moreover, the same authors showed that cognitive interference in a “Stroop interference task” can be mitigated by consonant music and potentiated by dissonant music (Masataka and Perlovsky, 2013). Both findings suggest that music, when appreciated, might provide the necessary aesthetic reward to tolerate conflicting cognitive states and uncertainty (Masataka and Perlovsky, 2013).

## Aesthetic Appreciation in the Perception–Action Cycle: Evidence From Neuroimaging

The previously described knowledge-oriented (Biederman and Vessel, 2006) “aesthetic attitude” prompted by the expectation of aesthetic rewards (e.g., musical pleasure; Ferreri et al., 2019) is related to specific brain activations subserving the link between aesthetic emotions and knowledge acquisition (Schoeller and Perlovsky, 2016; Sarasso et al., 2020a).

More specifically, recent neurocomputational models suggest that aesthetic appreciation may represent the conscious feedback of successful minimization of prediction errors *via* the update of predictive representations of the environment (Schmidhuber, 2009; Van de Cruys and Wagemans, 2011; Schoeller and Perlovsky, 2016; Sarasso et al., 2020a). In other words, aesthetic pleasure arises in correspondence with the improvement of the predictions about the incoming sensory stimulation. In the case of music, sounds might become aesthetically rewarding because of the refinement of musical expectations (Hansen et al., 2017; Koelsch et al., 2019). This learning-driven aesthetic reward has been shown to be mediated by the frontal dopaminergic network (Ferreri et al., 2019). Neurophysiologically, the pattern of activation of these circuits overlaps with that elicited by informational gains (Schwartenbeck et al., 2016) and the refinement of representational models (Mencke et al., 2019) that are involved in music perception (Koelsch et al., 2019; Mencke et al., 2019). The aesthetic dopaminergic reward may therefore constitute the intrinsic motivation to learn something new (Ferreri et al., 2019), thus helping the individual to tolerate the risk arising from sensory and cognitive uncertainty and to focus on learning-oriented activities (i.e., refining mental predictive sensory models; Koelsch et al., 2019; Mencke et al., 2019).

Moreover, aesthetic appreciation correlates with enhanced activations in early sensory areas (including mirror activations; Nadal, 2013; Sarasso et al., 2019, 2020b) and motor inhibition (see Sarasso et al., 2020a for a review). During the perception of more appreciated musical sounds, as an example, automatic defensive motor responses to surprising (i.e., uncertainty arising) stimuli are inhibited (Brattico et al., 2013). While increased sensory activations are thought to reflect a knowledge-oriented (Biederman and Vessel, 2006) attentional focusing (Vartanian and Goel, 2004; Nadal, 2013) on the object perceptual features, motor inhibition is crucial to slow down action production (Gallese, 2017; Sarasso et al., 2020a). In other words, transient states of motor inhibition free resources to update sensory representations in response to unexpected events (Wessel and Aron, 2017). Within a predictive coding framework, beauty might induce our brain to momentarily minimize prediction errors through representations update rather than action production. The suspension of previously acquired prototypical actions allows the planning of new motor responses on the basis of newly-acquired information (Sarasso et al., 2020a) and, at a phenomenological level, makes room for more intense emotions and sensations (Menninghaus et al., 2017). As Vittorio Gallese writes: “immobility, that is, a greater degree of motor inhibition, probably allows us to allocate more neural resources, intensifying the activation of bodily-formatted representations, and in so doing, making us adhere more intensely to what we are simulating” (Gallese, 2017, p. 48).

Such an emotional amplification triggered by musical aesthetic appreciation might involve the collective ability to learn and adapt (Bericat, 2016), especially when fast collective behavioral updates are vital. Group-level emotions are powerful predictors of policy support and guide social change (Halperin et al., 2013). Collective emotions accompany social action, and as evidenced by recent developments in social neuroscience, collective decisions often rest on emotional contagion (Bosse et al., 2013). Affect and emotions represent fast and parsimonious ways of representing the world in uncertain, complex situations, thereby guiding judgement, decision-making, and adaptive action (Damasio, 1996).

## Discussion

In summary, we believe that arts are not just hobbies, but as Hillman (1988) proposes, activities that might “challenge collective anesthesia.” According to the author, without this artistic function, we would become insensible toward each other and emotionally numb with respect to our environment. According to this view, the artistic experiences shared by people from all over the world during the lockdown for the Covid-19 pandemic, rather than representing simple folkloric manifestations, might have served a specific social adaptation function. We propose, as a preliminary hypothesis, that the social sharing of emotions conveyed by music can help to tolerate and amplify novel uncertainty-arising affective signals, which in turn would enable the adaptive update of behaviors and beliefs (Eyerman and Jamison, 1995). This idea needs further experimental confirmation and is still lacking a unified understanding of the scientific results from various disciplines, from low-level sensory processes to higher and more complex social phenomena. At the level of collective decision-making and adaptive change, the role of music should be further analyzed under the hypothesis of a twofold effect: (a) the enhancement of interoceptive awareness of affective visceral states prompted by the sharing of emotions through communication tools, such as music (Liljeström et al., 2013), and (b) the reduction of the dissonance between conflicting attitudes, emotions, and cognitions (Masataka and Perlovsky, 2012), coherently with the hypothesized role of aesthetic emotions in our ability to tolerate uncertainty for the sake of knowledge acquisition (Sarasso et al., 2020a). Novel neuroscientific findings at the level of sensory cognition suggest that musical aesthetic emotions might allow us to better attune to environmental changes. If this preliminary evidence is confirmed and extended to higher socio-cognitive levels by future research, public policies should regard artistic training as a crucial educational activity that might shape more “open” societies.

## Author Contributions

PS and IR wrote the article. MN-M and KS reviewed the article. All authors contributed to the article and approved the submitted version.

## Conflict of Interest

The authors declare that the research was conducted in the absence of any commercial or financial relationships that could be construed as a potential conflict of interest.
